# Supplementary material for: The prevalence and demographic features of congenital cytomegalovirus infection in an urban area of East Asia: A population-based study
Source: PLoS One. 2021 Mar 25;16(3):e0248801. doi: 10.1371/journal.pone.0248801 (PMC7993615; doi:10.1371/journal.pone.0248801)
Supplement: Attachment — Submitted filename: Response to reviewers_20201128_YTH1129_1212.docx [file pone.0248801.s002.docx]

Marta Giovanetti, Ph.D.

Academic Editor
PLOS ONE

November 28, 2020

Dear Dr. Giovanetti,

We thank you for the review of our manuscript and the opportunity to further improve our work (PONE-D-20-23918). We read with interest the comments made by the reviewers and we were pleased to revise our manuscript based on their comments. Following your guidance, we have carefully addressed the specific points raised by both reviewers. Appended at the bottom of this letter is an itemized, point-by-point reply to the reviewers’ comments. Meanwhile, we also attached a clean version of the revised manuscript with tracked changes accepted. We hope that this revised version can be accepted for publication in your prestigious Journal of *PLOS ONE.*

Thank you very much again for your kind consideration and valuable comments on our manuscript.

Sincerely yours,

Chun-Yi Lu, MD, PhD

Associate Professor

Department of Pediatrics

National Taiwan University Hospital, Taipei, Taiwan

and

Chen-Chi Wu, MD, PhD

Professor

Department of Otolaryngology

National Taiwan University Hospital, Taipei, Taiwan

**Journal Requirements:**

1. Our manuscript was prepared according to the PLOS ONE's style requirements, including those for file naming.
2. We would like to add "The study was supported by research grants from the National Taiwan University Hospital (107-S3913), Taipei City Government (10501-62-030), and the Ministry of Science and Technology, Taiwan (107-2622-B-002-008-CC2)" in the Funding Statement. We have removed the funding-related text from the manuscript.
3. We would like to state “The authors have declared that no competing interests exist."
4. Submit data is in attached file.

**Responses to Reviewer #1:**

The article presents a unique study focused on a locality (Taipei, Taiwan), aiming at a general and comprehensive background of East Asia. No details were given on the choice of the hospital to conduct the research. A more comprehensive study would be needed for the effect of cCMV infection in the population assessment.

**Response: We appreciate your valuable comment. We agree our study is not representative of the whole East Asia. Our study was conducted in an East Asia city, i.e. Taipei, Taiwan. To avoid confusions, we changed our title to “*The Prevalence and Hearing Outcome of Congenital CMV Infection in Taipei, Taiwan: Population-based Study*”.**

**As for the considerations behind the choice of the hospital to conduct the study, we have added explanations as the following (page 4):**

***“This hospital is the largest community hospital for maternal and child health care in the Taipei city, and provides service to the inhabitants of the whole Taipei metropolitan area. As high-risk pregnancy cases are usually referred to tertiary teaching hospitals, recruiting newborns from tertiary teaching hospitals may be prone to selection biases. As such, we chose the community-based Taipei City Hospital to conduct this study. Our previous study demonstrated that the epidemiological data collected from newborns of this hospital were representative of those of the Taiwanese population [Chu CW, Chen YJ, Lee YH, Jaung SJ, Lee FP, Huang HM. Government-funded universal newborn hearing screening and genetic analyses of deafness predisposing genes in Taiwan. Int J Pediatr Otorhinolaryngol. 2015;79(4):584-90. Epub 2015/03/01. doi: 10.1016/j.ijporl.2015.01.033. PubMed PMID: 25724631.].”***

The figure needs to improve resolution.

**Response: We have made the Figure with a high resolution of 300x300 dpi, which meets the requirement of the Journal. We are not sure if the resolution of the file viewed by the reviewer had been lowered to save space.**

**Responses to Reviewer #2:**

"The Prevalence and Hearing Outcome of Congenital CMV Infection in an Urban Area of East Asia: Population-based Study" by Yang et al. is well-written and provides a relatively comprehensive view about Congenital cytomegalovirus prevalence in Taipei, Taiwan. Although the study has limitations already recognized by the authors, this manuscript provide advances in our understanding about the relevance the early identification of cCMV in neonates increasing the quality of life of patients and better follow-up of the public service to these patients telling a nice story. I think it would be suitable for publication in PLoS One after careful revision of English language.

**Response: We appreciate your valuable comments. We do believe our study adds important information of congenital CMV infection, including its incidence and hearing outcome in Taipei, Taiwan. This will interest the PloS One readers. We have made our manuscript revised by a professional English editor.**
